# Psychometric validation of the French self and proxy versions of the PedsQL™ 4.0 generic health-related quality of life questionnaire for 8–12 year-old children

**DOI:** 10.1186/s12955-021-01714-y

**Published:** 2021-03-04

**Authors:** Pascal Amedro, Helena Huguet, Valerie Macioce, Raphael Dorka, Annie Auer, Sophie Guillaumont, Pascal Auquier, Hamouda Abassi, Marie-Christine Picot

**Affiliations:** 1 Pediatric and Congenital Cardiology Department, M3C Regional Reference Center, Arnaud De Villeneuve University Hospital, 371 Avenue du Doyen Giraud, 34295 Montpellier, France; 2 PhyMedExp, University of Montpellier, CNRS, INSERM, Montpellier, France; 3 Epidemiology and Clinical Research Department, University Hospital, Montpellier, France; 4 Clinical Investigation Center, INSERM-CIC 1411, University of Montpellier, Montpellier, France; 5 Pediatric Cardiology and Rehabilitation Center, Institut-Saint-Pierre, Palavas-Les-Flots, France; 6 Department of Public Health, Aix-Marseille University, EA 3279 Research Unit, Marseille, France

**Keywords:** Quality of life, Patient-reported outcome, Psychometric validation, Pediatrics, Kidscreen, Pediatric cardiology

## Abstract

**Background:**

The Pediatric Quality of Life Inventory Version 4.0 (PedsQL^TM^4.0) is a generic health-related quality of life (HRQoL) questionnaire, widely used in pediatric clinical trials but not yet validated in France. We performed the psychometric validation of the self and proxy PedsQL^TM^4.0 generic questionnaires for French children aged 8–12 years old.

**Methods:**

This bicentric cross-sectional study included 123 children and their parents with congenital heart disease (CHD) and 97 controls. The psychometric validation method was based on the consensus-based standards for the selection of health measurement instruments (COSMIN). The reliability was tested using the intraclass correlation coefficient (ICC). To evaluate the validity of this scale, content, face, criterion, and construct validity psychometric proprieties were tested. Acceptability was studied regarding questionnaires’ completion and the existence of a floor or a ceiling effect.

**Results:**

Test–retest reliability intra-class correlation coefficients were mainly in good range (0.49–0.66). Face validity was very good among parents (0.85) and children (0.75). Content validity was good (0.70), despite misinterpretation of some items. In construct validity, each subscale had acceptable internal consistency reliability (Cronbach's α > 0.72 in self-reports, > 0.69 in proxy-reports). In the confirmatory factor analysis, the goodness-of-fit statistics rejected the original structure with 4 factors. The exploratory factor analysis revealed an alternative two-factor structure corresponding to physical and psychological dimensions. Convergent validity was supported by moderate (> 0.41) to high correlations (0.57) between PedsQL and Kidscreeen questionnaires for physical, emotion and school dimensions. The ability of the PedsQL to discriminate CHD severity was better with physical, social and total scores for both self-reports and proxy-reports.

**Conclusions:**

The PedsQL^TM^4.0 generic self and proxy HRQoL questionnaires found good psychometric properties, with regard to acceptability, responsiveness, validity, and reliability. This instrument appeared to be easy to use and comprehend within the target population of children aged 8 to 12 years old and their parents.

*Trial registration*: This study was approved by the South-Mediterranean-IV Ethics Committee and registered on ClinicalTrials.gov (NCT01202916), https://clinicaltrials.gov/ct2/show/NCT01202916.

## Background

Quality of life (QoL) assessment in pediatrics has been given more attention in the past decade, although patient-reported outcomes (PRO’s) are not systematically quantified by caregivers and physicians, who primarily rely on clinical symptoms and disease complications [1, 2].

Nowadays, most medicine agencies recommend measuring PRO’s in pediatric drug trials [3, 4]. Quality of life is a general and subjective concept, which has been defined as the “overall life satisfaction” [5]. However, clinical trials require a more operational definition and use validated instruments with good psychometric properties [5, 6]. Those instruments, named “health-related quality of life” (HRQoL) questionnaires, are multidimensional and usually include physical and psycho-social aspects [6, 7].

The Pediatric Quality of Life Inventory Version 4.0 (PedsQL^TM^4.0) is a four-dimension HRQoL questionnaire widely used in pediatric clinical trials in healthy and chronically ill children [8–16]. Initially, the PedsQL^TM^4.0 generic core scale has been validated in a cohort of 963 American children [17]. Currently, self and proxy PedsQL questionnaires are available in various age and language versions [18–22]. Our group has increasingly used this instrument in HRQoL studies in France, among children with congenital heart disease (CHD) [14–16, 23–25]. However, no complete psychometric validation of the PedsQL has been performed yet in the French pediatric population.

Therefore, we aimed to perform the psychometric validation of the self and proxy PedsQL^TM^4.0 generic questionnaires for French 8–12 year-old children, from a cohort of subjects recruited in the general population and in tertiary care pediatric CHD centers.

## Methods

### Study design

This cross-sectional validation study was carried out between April 2013 and April 2016 (36 months) in pediatric patients with a congenital heart disease (CHD) and in children from the general population. Patients were prospectively recruited in two French tertiary care pediatric cardiology departments. The control children were recruited in 5 school classes (one per level from 3rd grade (elementary school) to 7th grade (middle school)), randomly selected in southern France (Occitanie Region) from the Education Ministry database.

### Study population

Children with a CHD aged 8–12 were prospectively recruited in the two participating centers during a pediatric cardiology outpatient visit. Inclusion procedures were beforehand harmonized. We did not include children with any other severe chronic disease (neurodevelopmental disorder, chronic renal or respiratory failures) and children and/or families unable to understand the questionnaire. The pediatric CHD population was stratified into 4 severity groups described by Uzark et al*.* [12].

In the control group, all children aged 8–12 and their parents, among the 5 selected school classes, were offered to participate in the study. The recruitment procedure was the same for each class and common to the one at the hospital.

### QoL questionnaires

Originally, the measurement properties of the PedsQL were analysed by Varni et al., who found an acceptable internal consistency reliability for group comparisons, in the total scale score (α = 0.88 child, 0.90 proxy), the physical health summary score (α = 0.80 child, 0.88 proxy), and the psychosocial health summary score (α = 0.83 child, 0.86 parent). The authors showed that the PedsQL could discriminate healthy and ill children and correlated with morbidity and illness burden. Cross-cultural validity of the PedsQL has shown similar properties to the original instrument in several countries [18, 20, 22, 26, 27].

The 8–12 year old self and proxy PedsQL^TM^4.0 generic HRQoL questionnaires have each four multidimensional scales: physical (8 items), emotional (5 items), social (5 items), and school (5 items) functioning. The three summary scores are the total score (23 items), the physical health summary score (8 items), and the psychosocial health summary score (15 items). To creat the psychosocial health summary score, the mean is computed as the sum of the items over the number of items answered in the emotional, social, and school functioning scales. The physical health summary score it the same as the physical functioning scale score [17].

Each item uses a 5-point Likert scale from 0 (never) to 4 (almost always). Items are reversed scored and linearly transformed to a 0–100 scale, higher scores indicating a better HRQoL.

**Fig. 1:**
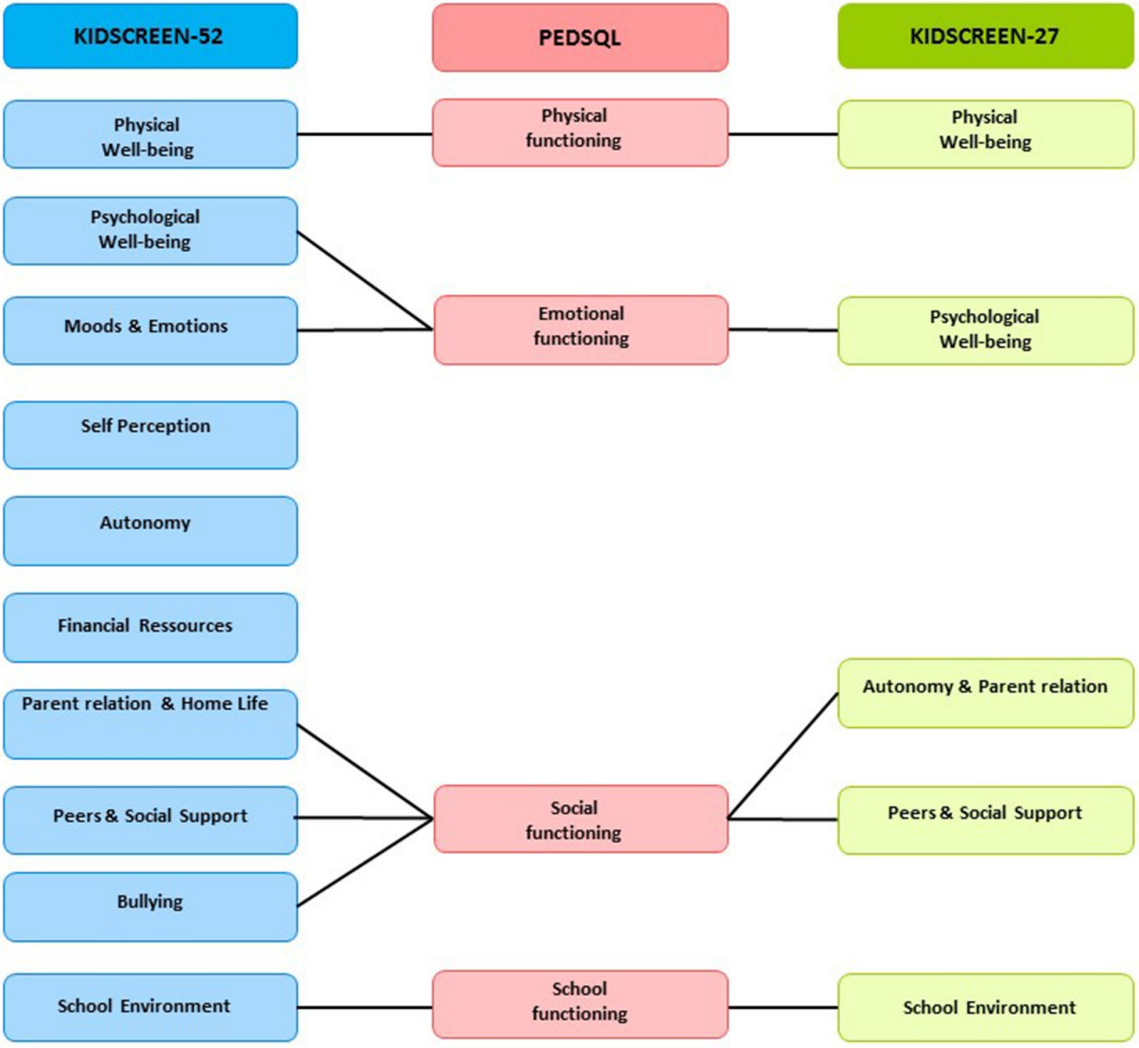
Correspondence between dimensions of the PedsQL, Kidscreen-52 and Kidscreen-27 quality of life questionnaires

The logistical process for filling in questionnaires was similar in both groups, as described in our previous studies [28, 29]:

- Children filled in the self-version of the 8–12 year old PedsQL^TM^4.0 generic questionnaire, under trained specialist nurse supervision, while parents filled in the proxy version in a separate room.
- At the same time, children and parents filled in separately the Kidscreen-52 (52 items) and the Kidscreen-27 (27 items) questionnaires, respectively [30, 31]. This European generic validated HRQoL instrument is designed for 8–18 y.o healthy and chronically ill children [30, 32]. We previously published Kidscreen self and parent-reported scores in CHD versus healthy children [28]. The dimensions of the Kidscreen and their correspondence with the PedsQL were reported in Fig. 1.

### Statistical analysis

A sample size of 124 CHD children was previously calculated for the pilot study, which aimed to analyse the relationship between CHD severity and the Kidscreen physical dimension [28].

In the control group, considering a recruitement in 5 school classes, with 30 children per class, and a 60% participation rate, we expected to include 90 control children. The total sample size (CHD and control children) therefore provides about 9 patients per item, which seems sufficient to perform factorial analysis and validate the PedsQL [33].

The study population was described with means and SD for quantitative variables and with frequencies and percentages for qualitative variables. Quantitative variables were compared with the parametric Student's t-test when the distribution was Gaussian, and with the Mann–Whitney test otherwise. Missing data were not substituted. Qualitative variables were compared with the chi-square test or Fisher's exact test. Data were analyzed using the SAS software version 9.1 (SAS Institute, Cary, NC). The two-sided significance level was 0.05.

## Psychometric validation method

**Fig. 2:**
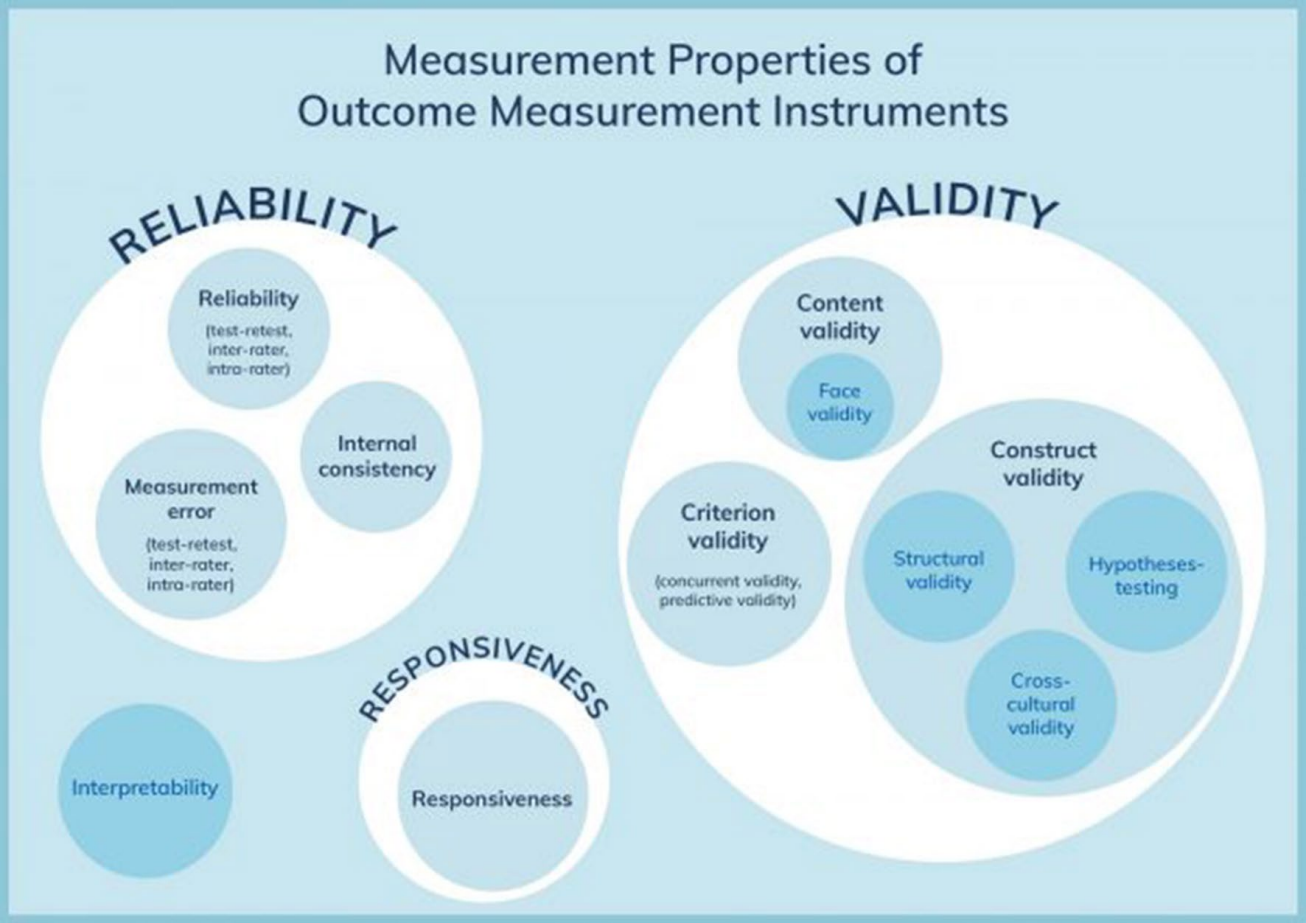
COSMIN taxonomy of relationships of measurement properties [34]. Abbreviations: COSMIN, COnsensus-based Standards for the selection of health Measrurement Instruments; HR-PRO, health-related patient reported outcome. Permission to reproduce this figure was obtained from Elsevier and Copyright Clearance Center for print and electronic format (License number: 4823570991456)

The psychometric validation method was based on the consensus-based standards for the selection of health measurement instruments (COSMIN) [34]. The COSMIN taxonomy of relationships of measurement properties was illustrated in Fig. 2.

### Reliability

Two weeks after first assessment, children and their parents filled in again at home the same PedsQL versions, and mailed them back to the study coordinator. In the CHD group, only patients with a stable clinical status, during the interim period, as assessed by their pediatric cardiologist (e.g. no modification in terms of medical treatment and no hospitalization), were included in the test–retest procedure. The reliability of the PedsQL was estimated with the intraclass correlation coefficient (ICC) and its 95% confidence interval (CI).

### Validity

#### Content validity

A group of five experts (specialist nurse, Ph.D. student in public health, adult expert patient with a CHD, and two pediatricians) assessed the simplicity and clarity of the questionnaire with a likert scale (1–4) ranging from unfavorable to favorable opinion, and evaluate whether items assess defined content, providing recommendations to add or remove any items. The content validity index (CVI) was defined as the number of experts who answered 3 or 4 divided by the total number of experts. CVI < 0.4, between 0.4 and 0.75, or > 0.75 indicated poor, intermediate-to-good, or excellent relevance, respectively [35, 36].

#### Face validity

A sample of 10 children and 10 parents read, answered and discussed each item during a face-to-face interview with the principal investigator. They gave their opinion on the scale layout, the length and the wording of the items and modalities of the answers. The investigator wrote down their answers and unclear items were reviewed [37, 38]. Items’ clarity was ranked 0 (not clear) or 1 (clear) [39]. The total number of points divided by the number of participants determined the face validity index.

#### Criterion validity

Correlation analyses were performed between PedsQL and Kidscreen dimensions, for both self and parents reports, to assess concurrent valididy.

#### Construct validity

##### Structural validity

In the multitrait-multi-item analysis, five hypotheses were tested. (1) Redundancy between items was assessed by calculating inter-items correlations within each dimension. Items of a given dimension were considered as non redundant if inter-items correlations were < 0.7. (2) Item-internal consistency (IIC) was assessed by correlating each item with its corresponding scale. An IIC was considered as satisfactory if 90% of the possible item-scale correlations were > 0.4. (3) Item discriminant validity (IDV) was assessed by determining to which extent the items correlated more with the dimensions they were supposed to reflect, than with any other dimensions. (4) Another assumption of the multi-trait analysis was that the coefficient of variation of each item is equal or higher than 20%. (5) The internal consistency validity reflected interrelations between PedsQL items, as assessed by the Cronbach α [40]. A value > 0.7 was considered as acceptable.

We first performed a confirmatory factor analysis (CFA) with 4 factors to test the original 4-factor structure in our population. Then, as the CFA did not find a good fit on the original structure, an exploratory factor analysis (EFA) was performed to optimize the quality of the fit and a 2-factor structure was tested. A CFA was then performed on the 2-factor structure.

In the CFA, we used a structural equation modeling, according to the 4 dimensions of the original PedsQL instrument, by fixing the variance of the latent constructs (factors) to 1.0, and leaving free the correlation between the latent constructs. The following absolute fit indices were calculated: the baseline model chi-square estimate (p χ^2^), the adjusted goodness-of-fit (AGFI, interpreted similar to an R2 estimate), the root mean square error of approximation (RMSEA, the closer to zero, the better the model fit), the standardized root mean square residual (SRMR, the closer to zero, the better the model fit) and the comparative fit index (CFI, preferable estimate is greater than 0.80). The goodness of fit of the model was considered as well-fitted if p_χ_^2^ > 0.05, RMSEA and SRMR < 0.08, and AGFI and CFI ≥ 0.80. The EFA was performed, using oblique rotation and polychoric correlations to identify the most appropriate factor structure. The number of factors was determined using scree test and parallel analysis (with 100 simulations). When the item’s factor loadings (in absolute value) were above one divided by the square root of the number of items, then the item was considered as being part of the factor. The variance explained by each factor (computed without taking the other factors into account) was calculated.

##### Hypothesis testing

The spearman correlation between the physical dimension of each instrument (Kidscreen and PedsQL) and the actual child’s physical capacity, as assessed by the maximum oxygen uptake (VO2_max_) during an exercise test, was calculated.

##### Cross cultural validity

The linguistic validation process from English to French was performed by MAPI institute, using a 4-step methodology: (1) forward translation step by 2 professional translators (reconciliation, quality control and discussion → target language version 1); (2) backward translation step by a professional translator (quality control and discussion → target language version 2); (3) adaptation step (review and adaptation of the mother language version to context of the target country → target language version 3); (4) cognitive debriefing step (on 3 parents of healthy children → final target language version) [41].

### Interpretability

#### Acceptability and quality of items

The questionnaires’ completion rate was reported. The existence of a floor effect (i.e. responses on the questionnaire cluster at the more negative health state end of the scale) or a ceiling effect (i.e. responses on the questionnaire cluster at the more positive health state end of the scale) was determined by the rate of children and parents who scored at the minimum (0) or maximum values (100), respectively for each item and dimension.

#### Discriminant validity

The PedsQL scores were compared between CHD and control populations, between girls and boys, and between four levels of disease severity [12]. For pairwise comparisons between each severity class, Holm’s correction was applied and the two-sided Jonckheere trend test investigated the existence of a trend according to this severity.

## Results

### Population

We included 220 children, of which 123 CHD and 97 controls. Among them, 210 children (117 CHD and 93 controls) completed the PedsQL self-questionnaire and 220 parents completed the PedsQL proxy-questionnaire.

Median age was 10 years (interquartile range 9–11 years), and 60% of children were boys. In the CHD group, severity class 1, 2, 3 and 4 concerned, respectively, 33 (27%), 14 (11%), 62 (50%) and 11 (9%) children.

### Psychometric validation

#### Reliability

Test–retest analyses showed that ICCs, overall and in each dimension, for both self and proxy reports, were in the range of 0.49 to 0.66, corresponding to moderate (0.41–0.6) to good agreement (0.6–0.8) (Table 1).

**Table 1:** PedsQL scores in CHD and control subjects: ceiling effect, effect size, internal consistency and reliability

	CHD patients					Controls					Mean difference (SD)	Effect size [95% CI]	Cronbach’s alpha	Retest reliability ICC [95% CI]
	N	Mean	SD	Range of items SD	% ceiling	N	Mean	SD	Range of items SD	% ceiling				
Self-reports														
Physical	117	80.9	16.2	[13.1; 32.39]	9.4	93	88.8	12.0	[6.6; 23.1]	20.4	7.88 (1.95) ***	0.55 [0.27; 0.82]	0.78	0.62 [0.53; 0.70]
Emotion	116	71.0	20.2	[23.7; 33.4]	6.0	93	76.7	18.0	[20.4; 32.4]	10.8	5.72 (2.65) *	0.30 [0.02; 0.57]	0.73	0.50 [0.39; 0.60]
Social	117	81.5	17.9	[22.1; 30.0]	21.4	93	89.1	16.6	[14.7; 24.4]	38.7	7.64 (2.39) ***	0.44 [0.16; 0.71]	0.79	0.57 [0.47; 0.66]
School	117	74.0	17.3	[21.0; 28.2]	5.1	93	83.0	15.7	[19.0; 26.2]	14.0	9.04 (2.28) ***	0.54 [0.27; 0.82]	0.72	0.61 [0.52; 0.69]
Psychosocial	117	75.4	15.3	NA	1.7	93	83.0	13.5	NA	6.5	7.56 (1.99) ***	0.52 [0.25; 0.80]	NA	0.61 [0.51; 0.69]
Total	117	77.3	14.2	NA	0.9	93	85.0	12.3	NA	6.5	7.67 (1.84) ***	0.57 [0.30; 0.85]	NA	0.66 [0.57; 0.73]
Proxy-reports														
Physical	123	79.3	20.0	[23.7; 32.1]	21.1	97	84.7	19.8	[22.5; 30.6]	22.7	5.38 (2.70) *	0.27 [0.01; 0.54]	0.86	0.49 [0.38; 0.59]
Emotion	123	63.8	21.3	[23.5; 32.3]	4.1	97	72.0	16.4	[22.0; 25.4]	3.1	8.2 (2.54) **	0.42 [0.16; 0.69]	0.81	0.64 [0.55; 0.71]
Social	123	79.0	19.1	[21.4; 32.5]	25.2	97	83.2	17.5	[15.5; 36.5]	32.0	4.23 (2.47)	0.23 [− 0.04; 0.49]	0.74	0.49 [0.38; 0.59]
School	123	68.9	19.0	[23.0; 33.6]	4.1	97	76.3	17.8	[15.1; 36.1]	4.1	7.48 (2.49) **	0.40 [0.13; 0.67]	0.69	0.60 [0.50; 0.68]
Psychosocial	123	70.6	16.0	NA	0.8	97	77.2	13.8	NA	0.0	6.62 (2.01) **	0.44 [0.17; 0.71]	NA	0.65 [0.56; 0.72]
Total	123	73.6	15.7	NA	0.8	97	79.8	14.3	NA	0.0	6.20 (2.02) **	0.41 [0.14; 0.68]	NA	0.63 [0.54; 0.71]

*CHD* congenital heart disease, *CI* confidence interval, *ICC* intraclass correlation coefficient, *NA* non applicable (range of items was not indicated in summary scores), *SD* standard deviation

% ceiling indicates the percentage of scores at the maximum value of the scaling range for each dimension

Mean difference = mean controls − mean CHD patients. *P-value < 0.05; **P-value < 0.01; ***P-value < 0.001

Effect size = mean difference ÷ pooled SD ([0.2; 0.5], small effect size; [0.51; 0.80], moderate effect size; > 0.80, large effect size)

#### Validity

##### Face validity and content validity

Face validity index was excellent in the parents’ group (0.85) and very good in the children group (0.75). However, for many children did not fully understand the meaning of item 4 (“it is hard for me to lift something heavy”), as most of them understood the question from a general perspective and not as a limitation potentially related to their health condition. During the interview, most children reported that “yes, it is hard for a child to lift something heavy, as compared to an adult”. Similarly, item 20 (“I forget things”) was frequently misunderstood, and two possible meanings were given for parents and children: forgetting concepts, lessons, or words during the class, or forgetting to bring an object to school (notebook, pencil case).

Content validity index was good (0.7). However, the experts considered that item 1 was not adapted to children living in the countryside (“It is hard for me to walk more than one block”). Moreover, item 4 (“It is hard for me to lift something heavy”) was often misinterpreted as mentioned before, and item 23 (“I miss school to go to the doctor or hospital”) was usually understood from a general perspective: both healthy and CHD children miss school to go to the doctor, but sick children may miss school more often than healthy subjects, which was not always interpreted this way.

##### Criterion validity

In terms of concurrent validity, PedsQL and Kidscreen corresponding dimensions correlated well in physical (r = 0.57), emotion (r = 0.49 with psychological well-being, 0.50 with moods and emotions, and 0.48 with self perception of the Kidscreen) and school dimensions (r = 0.41) for self-report (Table 2). For parents’ reports, these correlations were good in physical (r = 0.48), psychological (r = 0.57), and school (r = 0.49) dimensions. Indeed, the highest correlations observed between both instruments were those expected, except for social dimension. For the PedsQL social dimension, only one of the three corresponding dimensions of the Kidscreen (“bullying”) had a close-to-high correlation (r = 0.47). For parents reports, the social PedsQL dimension correlated better with the school dimension than with the two expected dimensions of the Kidscreen (“autonomy and parents relation” and “social support and peers”).

**Table 2:** Concurrent validity: correlation between PedsQL and Kidscreen 52 self-reports and between PedsQL and Kidscreen 27 parent-reports

	PedsQL questionnaire			
	Physical	Emotion	Social	School
*Self-reports (N = 199)*				
Kidscreen-52 questionnaire				
Physical well-being	**0.57**^**a,b**^	0.42	**0.40**	0.37
Psychological well-being	0.39	**0.49**^**a,b**^	0.31	0.27
Moods and emotions	0.39	**0.50**^**a,b**^	**0.40**	0.36
Self-perception	0.32	**0.48**	0.31	0.33
Autonomy	0.37	**0.45**	0.33	0.36
Parent relation and home life	0.28	0.35	0.27^a^	0.32
Financial resources	0.28	0.21	0.23	0.35
Social support and peers	0.25	0.30	0.29^a^	0.38
School environment	0.25	0.37	0.24	**0.41**^**a,b**^
Bullying	0.21	0.20	**0.47**^a.b^	0.26
*Proxy-reports (N = 203)*				
Kidscreen-27 dimensions				
Physical well-being	**0.48**^**a,b**^	0.43	0.35	0.32
Psychological well-being	0.24	**0.57**^**a,b**^	0.34	0.34
Autonomy and parent relation	− 0.03^NS^	0.23	0.11^NS,a^	0.14^NS^
Social support and peers	0.11 ^NS^	0.21	0.32^a^	0.26
School environment	0.09 ^NS^	0.31	0.37^b^	**0.49**^**a,b**^

Values are Spearman correlation coefficients (values ≥ 0.4 are marked in bold)

*NS* non-significant correlation (*P*-value > 0.05)

^a^Highest correlations expected for each PedsQL dimension

^b^Highest correlations observed for each PedsQL dimension

#### Construct validity

##### Structural validity

1. *Redundancy between items*
  In the PedsQL self-reports, none of the items had correlation coefficients above 0.70 for each dimension. In the proxy reports, strong correlations were found between items 2-running and 3-sports (r = 0.90, P < 0.001), and between items 19-attention and 21-schoolwork (r = 0.71, P < 0.001). All remaining items from the proxy reports had correlation coefficients < 0.70.
2. *Item-internal consistency (IIC)*
  Most correlations between items and the corresponding dimension were ≥0.4 (Additonal file 1: Table S1). Lower correlations were found for item 5-bath, 6-chores and 7-aches of the physical dimension (self-reports only), for item 17-doing-things of the social dimension for controls (self and proxy reports), and for item 22-feeling-well and 23-doctor of the school dimension for both CHD and control children.
3. *Item discriminant validity*
  In most cases, items correlated more with their own dimention than with other dimensions (Additonal file 1: Table S1). However, a few items better correlated with other dimensions, but with rather close correlation coefficients.
4. *Variability of items*
  Among CHD children, all items had a coefficient of variation above 20%, except item 5-bath, in self-reports only. Among control children, self-reports showed coefficients of variation < 20% for item 1-walking, 3-sports and 5-bath, and for item 18-playing. Parent-reports yielded coefficients of variation < 20% for item 17-doing-things, and items 22-not-feeling-well and 23-doctor.
5. *Internal consistency*
  In all 4 dimensions, Cronbach alpha coefficients were ≥0.69 (Table 1). Cronbach alpha coefficients for each dimension did not increase after removal of each item one by one.
6. *Factor analysis*
  The goodness-of-fit statistics of the confirmatory factor analysis indicated that the original structure with 4 factors may not be the best fit (for self-reports: p_χ_^2^ < 0.0001, AGFI = 0.698, RMSEA = 0.100 [0.092 ;0.109]_90%_, CFI = 0.731, and SRMR = 0.089; and for proxy reports: p_χ_^2^ < 0.0001, adjusted GFI = 0.611, RMSEA = 0.120 [0.112 ;0.128]_90%_, CFI = 0.724, and SRMR = 0.125. Indeed, for both reports, p_χ_^2^ were < 0.05, RMSEA and SRMR were < 0.08, and AGFI and CFI were < 0.80.

In the exploratory factor analysis, for self and proxy reports, only 2 factors were retained according to scree test and parallel analysis (Additonal file 2: Fig. S1), corresponding to physical (factor 1) and psychological (factor 2) dimensions. The factor loadings matrix and the variance explained by each factor were reported in Table 3.

**Table 3:** Two-factor loadings exploratory factor analysis

Item description	Item keyword	Self-reports		Proxy-reports	
		Factor 1	Factor 2	Factor 1	Factor 2
Variance explained		3.84	3.28	2.30	4.15
Physical dimension					
1 Walking more than one block	Walking	***0.81***	− 0.06	***0.99***	− 0.09
2 Running	Running	***0.95***	− 0.15	***0.79***	0.15
3 Participating in sports activity or exercise	Sports	***0.88***	− 0.09	***0.84***	0.10
4 Lifting something heavy	Lifting	***0.61***	0.15	***0.74***	0.10
5 Taking a bath or shower by him/herself	Bath	***0.67***	0.04	***1.01***	− *0.26*
6 Doing chores around the house	Chores	***0.27***	*0.22*	***0.70***	− 0.07
7 Having pain	Aches	*0.26*	***0.37***	0.20	***0.46***
8 Lack of energy	Energy	***0.55***	0.20	*0.21*	***0.55***
Emotion dimension					
9 Being afraid	Afraid	0.20	***0.47***	− 0.05	***0.72***
10 Feeling sad or blue	Sad	*0.25*	***0.50***	0.02	***0.80***
11 Feeling angry	Angry	− 0.10	***0.73***	− 0.11	***0.74***
12 Trouble sleeping	Sleeping	− 0.06	***0.54***	− 0.03	***0.72***
13 Worrying about what will happen next	Future	*0.37*	***0.43***	− 0.14	***0.82***
Social dimension					
14 Getting along with other children	Getting along with kids	0.12	***0.74***	***0.60***	0.12
15 Other kids refusing to be friends	No friends	− 0.04	***0.76***	0.07	***0.55***
16 Getting teased by other children	Teased	− 0.09	***0.82***	0.00	***0.66***
17 Not able to do things that other children his or her age can do	Doing things	***0.71***	− 0.03	*0.30*	***0.54***
18 Keeping up when playing with other children	Playing	***0.78***	0.12	***0.84***	0.06
School dimension					
19 Paying attention in class	Attention	0.01	***0.61***	***0.55***	0.06
20 Forgetting things	Forgetting	0.00	***0.73***	− 0.02	***0.57***
21 Keeping up with schoolwork	Schoolwork	*0.25*	***0.51***	***0.66***	0.06
22 Missing school because not feeling well	Not feeling well	***0.58***	− 0.02	***0.36***	*0.34*
23 Missing school to go see the doctor	Doctor	***0.45***	0.10	0.14	***0.43***

Values marked in italic represent items participating to each factor; values marked in bold represent highest factor loadings for each item.

In the PedsQL self-questionnaire, factor 1 included all items from the physical dimension, except one item (item 7-aches), and also included some items from the social dimension that could be interpreted by children as physical actions (items 17-doing-things and 18-playing), as well as two items from the school dimension, referring to somatic problems (items 22-not-feeling-well and 23-doctor). In the PedsQL proxy-questionnaire, factor 1 included all items of the physical dimension, but two items (items 7-aches and 8-energy), which were considered as belonging to the psycho-social domain. The factor 1 was therefore considered as the “physical and health dimension”. The factor 2, in both self and proxy-questionnaires, included most items in a psycho-social domain grouping psychological, emotion, social and school dimensions (Table 3), so it was considered as the “emotional and psychosocial dimension”. The confirmatory factor analysis (CFA) with these 2 factors found a slightly better goodness-of-fit statistics (Additonal file 1: Table S3). In the 4-factor loadings analysis, most items of the self-questionnaire were grouped in factor 1 for the physical dimension, factor 2 for the emotional and social dimensions, and factor 4 for the school dimension. As the proxy-questionnaire, most items could be grouped in factor 1 for the physical dimension, factor 2 for the emotional dimensions, factor 3 for the social dimension, and factor 4 for the school dimension (Additonal file 1: Table S2).

##### Hypothesis testing

The original physical dimension of the PedsQL moderately correlated with physical capacity, as assessed by the VO2_max_, in both self-reports (r = 0.22, *P* = 0.08) and proxy reports (r = 0.35, *P* = 0.01). In the same patients, the correlations between the physical dimension of the Kidscreen and the VO2_max_ were even lower in both self-reports (r = 0.19, *P* = 0.16) and proxy reports (r = 0.25, *P* = 0.05).

#### Interpretability

##### Acceptability and quality of items

Among the 210 children who completed the PedsQL self-questionnaire, 98% had no missing items. As for the PedsQL proxy questionnaires, 213 of 220 parents (97%) had no missing items. Missing data did not relate to any specific item. Ceiling effect exceeded 20% for the social dimension (self and proxy reports for CHD and control children) and physical dimension (self-reports for CHD children and self and proxy reports for controls) (Table 1). Floor effect was 0% for all dimensions in both groups. At the item level, a high ceiling effect (≥ 80%) was observed for item 1-walking and item 5-bath of the physical dimension in CHD and control self and parent-reports, and in item 18-playing of the social dimension for control self-reports only. No significant floor effect was observed.

##### Discriminant validity

PedsQL self-reported scores were significantly lower in CHD children than in controls in all dimensions (Table 1). Effect size was medium for school, physical, psychosocial and total scores, and small for emotion and social scores. Parents-reported scores were lower for CHD patients in all dimension except the social one, with small effect sizes.

Differences in PedsQL scores by gender and CHD severity were reported in Table 4. Female self-reported HRQoL scores were lower than male’s scores for emotional, physical, and total scores. No difference was observed between boys and girls according to parents-reports. PedsQL self-reports were significantly different in terms of CHD severity for physical, social, psychosocial and total scores. PedsQL proxy-reports were significantly different in terms of CHD severity for physical, social, and total scores. The ability to discrimate CHD severity with the PedsQL was mainly observed, for both self and proxy questionnaires, between the low severity class (class 1) and the three other severity classes (2, 3 and 4), but not between the 2 intermediate severity classes (2 and 3).

**Table 4:** PedsQL scores according to gender and CHD severity

	Girls	Boys	*P*-value	Severity class 1	Severity class 2	Severity class 3	Severity class 4	*P*-value	Pairwise comparisons^a^	Trend test *P*-value^b^
*Self-reports*										
N	83	127		33	14	58	9			
Physical	82.0 (16.0)	85.9 (14.2)	**0.05**	86.6 (11.0)	79.5 (19.0)	80.6 (15.7)	62.9 (21.5)	**0.01**	1, 3 > 4	**0.01**
Emotion	70.0 (20.2)	75.9 (18.6)	**0.03**	73.5 (18.3)	58.6 (24.8)	72.6 (20.9)	72.8 (12.0)	0.16		0.84
Social	84.1 (19.1)	85.4 (16.8)	0.69	88.8 (14.1)	73.6 (27.6)	80.5 (16.5)	71.1 (14..5)	**0.01**	1 > 3, 4	** < 0.01**
School	75.9 (17.0)	79.3 (17.2)	0.09	79.4 (13.7)	67.1 (23.6)	72.2 (17.3)	76.1 (15.4)	0.15		0.14
Psychosocial	76.7 (15.2)	80.1 (14.8)	0.08	80.6 (11.0)	66.4 (23.0)	74.9 (15.4)	73.3 (8.5)	**0.03**	1 > 2	0.10
Total	78.5 (14.2)	82.1 (13.7)	**0.03**	82.7 (8.9)	71.0 (20.8)	76.9 (14.4)	69.7 (12.4)	**0.04**	1 > 2, 4	**0.03**
*Proxy-reports*										
N	88	132		33	14	62	11			
Physical	79.9 (20.3)	82.9 (19.9)	0.18	84.9 (18.8)	86.2 (20.2)	77.5 (19.7)	69.3 (18.9)	**0.01**		** < 0.01**
Emotion	67.9 (18.7)	67.0 (20.4)	0.83	68.0 (20.6)	62.9 (24.5)	62.0 (21.6)	65.9 (18.0)	0.68		0.46
Social	80.6 (17.6)	81.1 (19.1)	0.59	85.0 (21.1)	81.1 (19.1)	77.8 (17.8)	70.5 (17.0)	**0.02**	1 > 3, 4	** < 0.01**
School	73.1 (19.1)	71.5 (18.7)	0.50	73.3 (20.4)	71.1 (22.9)	66.5 (16.7)	70.5 (21.9)	0.23		0.14
Psychosocial	73.9 (15.0)	73.2 (15.7)	0.84	75.5 (16.8)	71.7 (19.5)	68.8 (14.8)	68.9 (14.8)	0.18		**0.05**
Total	76.0 (15.3)	76.6 (15.4)	0.68	78.7 (15.8)	76.7 (17.7)	71.8 (14.7)	69.1 (14.9)	**0.05**	1 > 3	**0.01**

Values are means (SD)

^a^Pairwise comparisons significantly different after Holm’s correction (*P*-value < 0.10) are marked in bold

^b^Significant comparisons with two-sided Jonckheere trend test are marked in bold.

## Discussion

In this study, from a cohort of 220 children, we analysed the psychometric properties of the self and proxy PedsQL™ 4.0 generic questionnaires for French children aged 8–12 years.

In a standardized test–retest procedure, we found a moderate to good reliability, overall and in each dimension, for both self and proxy reports. With an ICC of 0.66 for the self-reported total scores, the reproductibility of the PedsQL can be considered as good in this young pediatric population.

Face validity index was excellent in the parents’ group (0.85) and very good in the children group (0.75). However, two items had unexpected interpretations: for item 4 “is it difficult to lift something heavy?”, we suggest adding “compared to other children of your age”, to avoid any misunderstanding; and item 20 was understood in two different ways (forgetting to bring some objects at school or forgetting the lessons that have been learned), nevertheless, in both cases, the question intends to assess some degree of cognitive disorder.

Content validity index was good (0.7). However, item 1 should be adapted to children who don’t live in a city (“walk more than one block” could be replaced by “walk around the playground at school”), and item 23 (“I miss school to go to the doctor or the hospital”) could be separated in 2 questions (“do you miss school?” and “do you go to the doctor or hospital?”).

In terms of criterion validity, PedsQL and Kidscreen corresponding dimensions correlated well in physical, emotion and school dimensions, for both self and proxy reports.

In terms of construct validity, most items were not redundant, excepted for items 2 (running) and 3 (sports) of the physical dimension. Nevertheless we believe that both items are of interest as they may have different meanings in children concerned with some degree of sports restriction, such as in inherited cardiac arrhythmia: such children may be allowed to “run” in their everyday recreational physical activity, but suffer from competitive sports restriction [42]. Item-internal consistency was correct, nevertheless some items reflected more autonomy than physical well-being (items 5-bath, 6-chores), or were not appropriate in chronic disease not concerned with pain (item 7-aches). As a result, despite overall good item discriminant validity, those same items better correlated with another dimension than their own (e.g. social dimension for “aches” instead of physical dimension). Items variability was good, except for the poorly understood items. Nevertheless, we observed an acceptable internal consistency for each dimension (Cronbach alpha ≥ 0.69 in all dimensions). Interestingly, the confirmatory analysis did not bring out the original 4-factor structure of the PedsQL [17]. Therefore, we performed an exploratory analysis, which showed that a 2-factor structure seemed the most appropriate to summarize the information. However, those 2 factors did not fully correspond to the 2 original PedsQL sub-scores (i.e. physical and psycho-social). Indeed, factor 1 included all physical items as well as some items considered as physical by the children (item 17-doing things, item 18-playing, item 22-not feeling well, and item 23-doctor). Cross-cultural comparisons to the factor structure obtained in the original PedsQL publication have shown heterogeneous results, from 2 factors [43] to 5 factors [27]. Such comparisons should be interpreted with caution as the populations are different in terms of culture, clinical status and age range. Nevertheless, such findings may be of interest in clinical trials study design using dichotomized HRQoL scores to assess PRO as primary outcome, secondary outcome or even in composite scores. Therefore, the total score may be more appropriate in pediatric trials using the PedsQL [16].

We observed a moderate correlation between physical well-being assessed by the PedsQL and the actual physical capacity of the child, in both CHD and control groups. Interestingly, similar results were observed with the Kidscreen instrument and this correlation was better from parents reports with both instruments [29]. Indeed, the concept of quality of life is much broader than what VO2_max_ represents in healthy children and in children with a cardiac disease.

The acceptability of the self and proxy PedsQL instruments was excellent, with only 2% and 3% missing items, respectively. As in the original psychometric analysis of the PedsQL, no floor effect was observed [17]. However, a ceiling effect was observed in both CHD and control children, especially in the physical and social dimensions. A similar effect has been observed in the psychometric validation of the PedsQL from a large cohort of school children [44].

The PedsQL instrument provided a good discriminant validity, as all scores were significantly lower in CHD children than in controls, overall, in each dimension, and in both self and proxy reports (except for the parents reported social dimension). Moreover, the PedsQL could discriminate severe from non severe CHD, but was less performant to discriminate intermediate severity levels. Interestingly, gender differences were observed in self-reports, female HRQoL scores being lower than males in most dimensions, but not in parents reports. Gender differences have been commonly observed in pediatric HRQOL studies [21, 28]. For example, in boys with CHD, the feeling of overall well-being is linked to the practice of a physical activity, which is reflected in HRQOL scores [28, 29, 45]. A possible cofounding effect of gender on HRQOL’s perception may exist, however the impact of diferential item functioning using the PedsQL has been considered negligible [46, 47].

As classically observed in HRQoL studies in the pediatric population, our results highlighted the existing difference between self and parents reports, both in healthy and CHD children [24, 28]. Usually, proxy reports provide lower scores than self-reports, and, in our experience, parents’ reports seem to better reflect the actual disease severity, especially mothers of children with CHD [15, 25, 29].

### Study limitations

Response to clinical change was not assessed in this study. We previously found a good response to change with the French proxy-version of the PedsQL from a large cohort of mothers of children under oral anticoagulants [15]. Nevertheless, futher studies using PedsQL self reports to determine reponse to clinical change remain necessary. As it is our area of expertise, the CHD population was used to validate the PedsQL in this study, which made relevant the use of exercise capacity outcomes [45]. The lack of heterogeneity of the population may explain the moderate correlation of the PedsQL with disease severity [25, 29].

## Conclusion

The French version of the PedsQL^TM^4.0 generic self and proxy HRQoL questionnaires found acceptable to good psychometric properties, with regard to acceptability, responsiveness, validity, and reliability. This instrument appeared to be easy to use and comprehend within the target population of children aged 8–12 and their parents.

## Supplementary Information

**Additional file 1. Table S1**: Correlation between each PedsQL item and the score of its dimension without this item. **Table S2**: Four-factor loadings exploratoty factor analysis. **Table S3**: Confirmatory factor analysis with one-, two-, and four-factor structures.

**Additional file 2. Fig. S1**: Scree plot and parallel analysis in self-reported PedsQL scores.

## Data Availability

All data generated or analysed during this study are included in this published article. The datasets used for those published data are available from the corresponding author on reasonable request.

## Abbreviations

AGFI: Adjusted goodness-of-fit
CFI: Comparative fit index
CHD: Congenital heart disease
CI: Confident interval
CVI: Content validity index
HRQoL: Health related quality of life
ICC: Intraclass correlation
IDV: Item discriminant validity
IIC: Item internal consistency
PedsQL: Pediatric Quality of Life Inventory
PRO: Patient-reported outcomes
QoL: Quality of life
RMSEA: Root mean square error of approximation
SD: Standard deviation
SRMR: Standardized root mean square residual
VO2_max_: Maximum oxygen uptake
