# Supplementary material for: Psychometric validation of the French self and proxy versions of the PedsQL™ 4.0 generic health-related quality of life questionnaire for 8–12 year-old children
Source: Health Qual Life Outcomes. 2021 Mar 4;19:75. doi: 10.1186/s12955-021-01714-y (PMC7934389; doi:10.1186/s12955-021-01714-y)
Supplement: Supplementary file 1 — Additional file 1. Table S1: Correlation between each PedsQL item and the score of its dimension without this item. Table S2: Four-factor loadings exploratoty factor analysis. Table S3: Confirmatory factor analysis with one-, two-, and four-factor structures. [file 12955_2021_1714_MOESM1_ESM.docx]

**Supplementary Table 1. Correlation between each PedsQL item and the score of its dimension without this item**

|  |  | | CHD | | Controls | |
| --- | --- | --- | --- | --- | --- | --- |
| PedsQL item description | |  | Self-report | Proxy-report | Self-report | Proxy-report |
| Physical dimension | | | | | | |
| 1 | Walking more than one block |  | **0.5** | **0.6** | **0.4** | **0.6** |
| 2 | Running |  | **0.6** | **0.8** | **0.5** | **0.6** |
| 3 | Participating in sports activity or exercise |  | **0.6** | **0.8** | **0.4** | **0.7** |
| 4 | Lifting something heavy |  | **0.4** | **0.6** | **0.5** | **0.6** |
| 5 | Taking a bath or shower by him/herself |  | **0.3** | **0.5** | 0.1^d^ | **0.5** |
| 6 | Doing chores around the house |  | 0.2^b^ | **0.4** | 0.2^c^ | **0.4** |
| 7 | Having pain |  | 0.3^c^ | **0.5** | **0.6** | **0.6** |
| 8 | Lack of energy |  | **0.5** | **0.6** | 0.5^b^ | **0.4** |
| Emotion dimension | | | | | | |
| 9 | Feeling afraid or scared |  | **0.5** | **0.7** | 0.4^a^ | **0.4** |
| 10 | Feeling sad or blue |  | **0.6** | **0.6** | **0.5** | **0.5** |
| 11 | Feeling angry |  | **0.5** | **0.5** | **0.5** | **0.6** |
| 12 | Trouble sleeping |  | **0.4** | **0.6** | **0.4** | **0.5** |
| 13 | Worrying about what will happen to him/her |  | **0.5** | **0.6** | 0.5^a^ | **0.5** |
| Social dimension | | | | | | |
| 14 | Getting along with other children |  | **0.5** | **0.5** | **0.5** | **0.7** |
| 15 | Other kids refusing to be friends |  | **0.4** | **0.5** | **0.6** | **0.5** |
| 16 | Getting teased by other children |  | **0.6** | **0.5** | **0.5** | **0.5** |
| 17 | Not able to do things that other children his or her age can do |  | 0.4^a^ | **0.7** | 0.3^d^ | 0.3^b^ |
| 18 | Keeping up when playing with other children |  | 0.6^a^ | 0.6^a^ | **0.4** | **0.7** |
| School dimension | | | | | | |
| 19 | Paying attention in class |  | **0.4** | **0.6** | **0.5** | **0.7** |
| 20 | Forgetting things |  | **0.5** | **0.5** | **0.5** | **0.4** |
| 21 | Keeping up with schoolwork |  | **0.6** | **0.6** | **0.5** | **0.7** |
| 22 | Missing school because of not feeling well |  | **0.3** | 0.3^a^ | **0.3** | **0.3** |
| 23 | Missing school to go to the doctor or hospital |  | **0.3** | **0.3** | **0.4** | 0.2^a^ |

Correlation coefficients are marked in bold when the item best correlated with the score of its own dimension without this item.

Legend: ^a^ best correlated item with the physical dimension; ^b^ best correlated item with the emotion dimension; ^c^ best correlated item with the social dimension; ^d^ best correlated item with the school dimension.

Comments: In the CHD group, the item 6 of the physical dimension (“chores”) better correlated with the emotional dimension, the item 7 of the physical dimension (“aches”) better correlated with the social dimension, and the item 17 of the social dimension (“doing things”) better correlated with the physical dimension. In the control group, the item 5 of the physical dimension (“bath”) better correlated with the school dimension, the item 6 of the physical dimension (“chores”) better correlated with the social dimension, and the item 17 of the social dimension (“doing things”) better correlated with the school dimension.

**Supplementary Table 2. Four-factor loadings exploratoty factor analysis**

| **Item description** | **Self-reports** | | | |  | **Proxy-report s** | | | |
| --- | --- | --- | --- | --- | --- | --- | --- | --- | --- |
|  | **F1** | **F2** | **F3** | **F4** |  | **F1** | **F2** | **F3** | **F4** |
| **Physical dimension** | | | | | | | | | |
| 1 Walking more than one block | ***0.51*** | 0.20 | *0.39* | *-0.24* |  | ***0.92*** | -0.01 | 0.01 | 0.12 |
| 2 Running | ***0.91*** | -0.06 | 0.09 | -0.04 |  | ***0.84*** | *0.26* | 0.04 | -0.10 |
| 3 Participating in sports activity or exercise | ***0.92*** | -0.13 | 0.02 | 0.12 |  | ***0.90*** | 0.17 | 0.07 | -0.10 |
| 4 Lifting something heavy | ***0.49*** | 0.13 | *0.21* | 0.09 |  | ***0.77*** | *0.22* | 0.01 | -0.07 |
| 5 Taking a bath or shower by him/herself | 0.18 | -0.05 | ***0.78*** | 0.14 |  | ***0.96*** | -0.13 | -0.06 | 0.08 |
| 6 Doing chores around the house | -0.12 | 0.20 | ***0.60*** | 0.05 |  | ***0.61*** | -0.15 | 0.13 | 0.20 |
| 7 Having pain | 0.13 | ***0.76*** | 0.05 | *-0.35* |  | *0.20* | ***0.48*** | 0.11 | 0.00 |
| 8 Lack of energy | ***0.61*** | *0.29* | -0.09 | -0.01 |  | 0.20 | ***0.68*** | 0.02 | -0.01 |
| **Emotion dimension** | | | | | | | | | |
| 9 Being afraid | 0.12 | ***0.56*** | 0.10 | -0.01 |  | 0.06 | ***0.51*** | *0.37* | -0.12 |
| 10 Feeling sad or blue | *0.28* | ***0.49*** | -0.03 | 0.12 |  | -0.08 | ***0.65*** | *0.28* | *0.22* |
| 11 Feeling angry | -0.04 | ***0.47*** | -0.02 | *0.38* |  | -0.11 | ***0.43*** | *0.42* | 0.10 |
| 12 Trouble sleeping | *0.21* | *0.27* | *-0.30* | ***0.39*** |  | 0.14 | *0.40* | ***0.49*** | -0.19 |
| 13 Worrying about what will happen next | *0.27* | ***0.58*** | 0.10 | -0.07 |  | -0.02 | *0.52* | ***0.46*** | -0.13 |
| **Social dimension** | | | | | | | | | |
| 14 Getting along with other children | 0.00 | ***0.66*** | 0.19 | 0.20 |  | *0.33* | -0.16 | ***0.30*** | *0.58* |
| 15 Other kids refusing to be friends | -0.10 | ***0.76*** | 0.08 | 0.10 |  | 0.14 | -0.16 | ***0.81*** | 0.09 |
| 16 Getting teased by other children | -0.07 | ***0.71*** | 0.00 | *0.24* |  | 0.06 | -0.09 | ***0.85*** | 0.12 |
| 17 Not able to do things that other children his or her age can do | ***0.79*** | -0.01 | -0.08 | 0.05 |  | *0.28* | *0.28* | ***0.39*** | 0.11 |
| 18 Keeping up when playing with other children | ***0.71*** | 0.09 | 0.16 | 0.12 |  | ***0.61*** | 0.13 | 0.00 | *0.41* |
| **School dimension** | | | | | | | | | |
| 19 Paying attention in class | 0.07 | 0.00 | 0.14 | ***0.77*** |  | 0.05 | 0.04 | -0.02 | ***0.89*** |
| 20 Forgetting things | 0.07 | *0.27* | 0.05 | ***0.62*** |  | *-0.24* | *0.23* | *0.38* | ***0.49*** |
| 21 Keeping up with schoolwork | 0.19 | -0.07 | *0.30* | ***0.72*** |  | 0.17 | 0.14 | -0.10 | ***0.84*** |
| 22 Missing school because of not feeling well | 0.10 | 0.05 | ***0.70*** | -0.06 |  | *0.25* | ***0.63*** | -0.18 | 0.12 |
| 23 Missing school to go to see the doctor | 0.06 | 0.00 | ***0.63*** | 0.14 |  | 0.01 | ***0.76*** | *-0.23* | 0.13 |

Legend: Values in italic represent item participating to factor. Values in bold represent highest factor loadings for each item.

Supplementary Table 3. Confirmatory factor analysis with one-, two-, and four-factor structures

|  | **p χ²** | **AGFI** | **RMSEA** | **CFI** | **SRMR** |
| --- | --- | --- | --- | --- | --- |
| **Self-reports : 4 factors** | <0.0001 | 0.698 | 0.100 [0.092 ;0.109]_90%_ | 0.731 | 0.089 |
| **Proxy-reports : 4 factors** | <0.0001 | 0.611 | 0.120 [0.112 ;0.128]_90%_ | 0.724 | 0.125 |
| **Self-reports : 2 factors** | <0.0001 | 0.726 | 0.096 [0.088 ;0.105]_90%_ | 0.747 | 0.084 |
| **Proxy-reports : 2 factors** | <0.0001 | 0.613 | 0.128 [0.120 ;0.136]_90%_ | 0.681 | 0.107 |
| **Self-reports : 1 factor** | <0.0001 | 0.658 | 0.113 [0.105 ;0.121]_90%_ | 0.650 | 0.090 |
| **Proxy-reports : 1 factor** | <0.0001 | 0.459 | 0.154 [0.147 ;0.162]_90%_ | 0.531 | 0.138 |
