# Supplementary figures and images for: Psychometric validation of the French self and proxy versions of the PedsQL™ 4.0 generic health-related quality of life questionnaire for 8–12 year-old children
Source: Health Qual Life Outcomes. 2021 Mar 4;19:75. doi: 10.1186/s12955-021-01714-y (PMC7934389; doi:10.1186/s12955-021-01714-y)

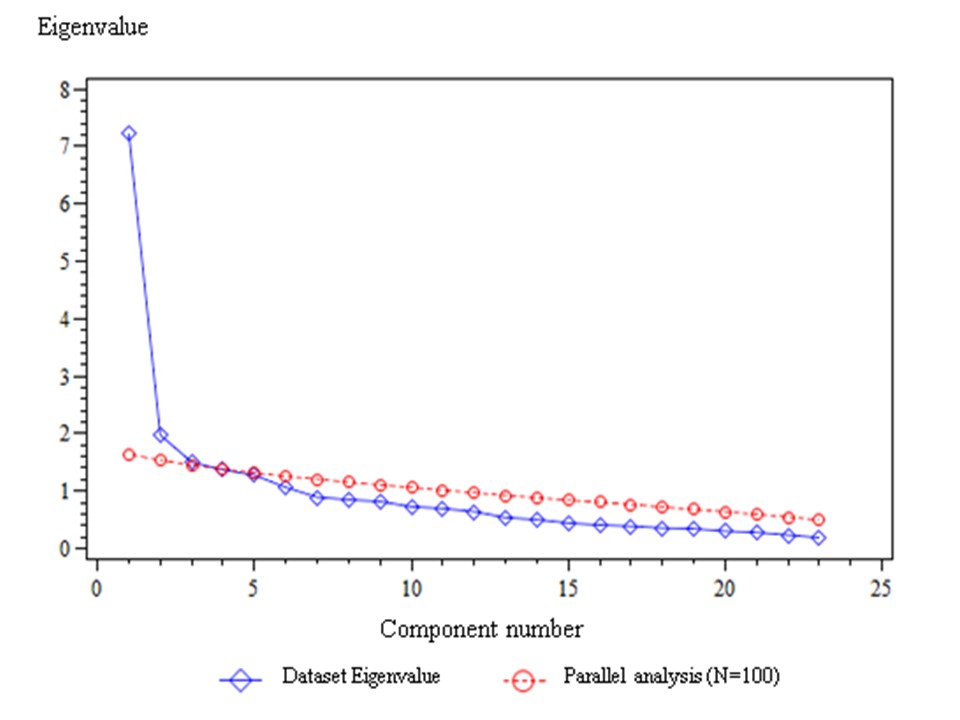

Supplement: Supplementary file 2 — Additional file 2. Fig. S1: Scree plot and parallel analysis in self-reported PedsQL scores. [file 12955_2021_1714_MOESM2_ESM.jpg]
